# Omega-3 Polyunsaturated Fatty Acids (n-3 PUFAs) for Immunomodulation in COVID-19 Related Acute Respiratory Distress Syndrome (ARDS)

**DOI:** 10.3390/jcm12010304

**Published:** 2022-12-30

**Authors:** Francesca Velotti, Lara Costantini, Nicolò Merendino

**Affiliations:** Department of Ecological and Biological Sciences (DEB), University of Tuscia, Largo dell’Università, 01100 Viterbo, Italy

**Keywords:** n-3 PUFAs, EPA, DHA, immunomodulation, COVID-19, SARS-CoV-2, ARDS, immune response, lung epithelial cells, clinical trials

## Abstract

Coronavirus disease-2019 (COVID-19), caused by severe acute respiratory syndrome-coronavirus 2 (SARS-CoV-2), might be complicated by Acute Respiratory Distress Syndrome (ARDS) caused by severe lung damage. It is relevant to find treatments for COVID-19-related ARDS. Currently, DHA and EPA n-3 PUFAs, known for their immunomodulatory activities, have been proposed for COVID-19 management, and clinical trials are ongoing. Here, examining COVID-19-related ARDS immunopathology, we reference in vitro and in vivo studies, indicating n-3 PUFA immunomodulation on lung microenvironment (bronchial and alveolar epithelial cells, macrophages, infiltrating immune cells) and ARDS, potentially affecting immune responses in COVID-19-related ARDS. Concerning in vitro studies, evidence exists of the potential anti-inflammatory activity of DHA on airway epithelial cells and monocytes/macrophages; however, it is necessary to analyze n-3 PUFA immunomodulation using viral experimental models relevant to SARS-CoV-2 infection. Then, although pre-clinical investigations in experimental acute lung injury/ARDS revealed beneficial immunomodulation by n-3 PUFAs when extracellular pathogen infections were used as lung inflammatory models, contradictory results were reported using intracellular viral infections. Finally, clinical trials investigating n-3 PUFA immunomodulation in ARDS are limited, with small samples and contradictory results. In conclusion, further in vitro and in vivo investigations are needed to establish whether n-3 PUFAs may have some therapeutic potential in COVID-19-related ARDS.

## 1. Introduction

COVID-19 is a respiratory-related disease caused by a highly pathogenic coronavirus known as SARS-CoV-2 [1]. In most cases, COVID-19 results as an asymptomatic or mild disease, whereas in around 10–20% of infected patients, it appears as a severe disease, complicated with severe lung damage called acute respiratory distress syndrome (ARDS), often lethal [2]. ARDS is characterized by diffuse pulmonary damage, consisting of permanent damage of alveolar epithelial cells and capillary endothelial cells, that leads to an acute and diffuse inflammatory injury into the alveolar-capillary barrier, which is associated with increased vascular permeability and reduced compliance, compromising thus gas exchange, and causing hypoxemia [3]. COVID-19-related ARDS occurs approximately between 9 and 12 days following the onset of symptoms and represents a major adverse event, leading to an overall mortality rate of 40% to 60% [4].

The introduction of vaccination against SARS-CoV-2 often cannot block infection but provides immunity to reduce severe disease and ARDS [5,6]. However, the vast difference in the vaccination percentage in different parts of the world allows virus diffusion and replication, leading to the emergence of new SARS-CoV-2 variants, causing from average to life-threatening pneumonia and ARDS. Accordingly, the public and health systems plan for the possibility that COVID-19 will persist and become a persistent seasonal disease [7]. Therefore, since the respiratory apparatus is a major target of SARS-CoV-2 and, to date, specific therapy for COVID-19-related ARDS does not exist, it is relevant and urgent to find treatments that, alone or as adjuvant therapies, prevent and/or cure this severe lung disease.

Omega-3 polyunsaturated fatty acids (n-3 PUFAs) consist of different compounds, of which the most important are found in flaxseed and fish, α-linolenic acid (ALA), eicosapentaenoic acid (EPA) and docosahexaenoic acid (DHA), and play an important role in the human diet and physiology [8,9]. Noteworthy, higher n-3 PUFA levels in the blood have been associated with a lower risk of mortality for different kinds of diseases [10].

Several investigations show that n-3 PUFAs exert immunomodulatory activity, either directly by targeting immune and non-immune cells or indirectly by targeting the gut microbiome [11,12,13,14]. In particular, EPA and DHA are known for their anti-inflammatory [11,12,13] and inflammation-resolving activities [15,16]. Indeed, these compounds can inhibit cell expression of pro-inflammatory cytokines and chemokines [11,13], as well as can be metabolized into specialized pro-resolving mediators (SPMs), such as resolvins, protectins, and maresins, that actively resolve inflammation (including those due to infections) [16]. Therefore, particular interest has arisen in n-3 PUFAs as potential therapeutics for both the prevention and treatment of human diseases associated with inflammation, including cancer and metabolic and cardiovascular diseases [17,18,19,20,21,22,23,24,25].

Currently, several investigators propose the use of n-3 PUFAs as antiviral and immunomodulatory agents for the management of COVID-19, its progression, and complications, including ARDS [26,27,28,29,30,31,32,33,34,35], and a certain number of clinical trials have been already approved for the use of n-3 PUFAs for the prevention (Table 1) [36,37,38] or the cure (Table 2) [37,39,40,41,42,43,44] of COVID-19 and its complications.

Most of these studies are ongoing, and the results are not yet available in a peer-reviewed publication. However, the VASCEPA-COVID-19 trial (Table 2) has been completed, and the published results provide evidence that oral administration of icosapent ethyl for 14 days in a modest (100) sample of outpatients with COVID-19 induced an early anti-inflammatory response (consisting in the significant reduction of high-sensitivity C-reactive protein-CRP) and an improvement of symptoms (assessed by using the InFLUenza Patient-Reported Outcome score) [40]. In addition, in another completed study and published paper, Doaei et al. reported that EPA plus DHA supplementation for 14 days in a very small (50) sample of critically ill COVID-19 patients improved the levels of several parameters of respiratory and renal function; however, no analysis was carried out on immunomodulation and its possible correlation with clinical outcomes [43].

In this review, we focus our attention on COVID-19-related ARDS immunopathology, referencing data in the literature that suggest a potential immunomodulatory activity of n-3 PUFAs on the pulmonary microenvironment, mostly composed of immune cells (e.g., alveolar macrophages, infiltrating monocytes and neutrophils) as well as bronchial and alveolar epithelial cells. In particular, we highlight the in vitro and in vivo immunomodulatory activity of DHA and EPA potentially affecting the immunological biomarkers associated with COVID-19-related ARDS, whereas we refer to other reviews for the potential direct antiviral activity of n-3 PUFAs, including the inhibition of SARS-CoV-2 replication and specific receptor binding [45,46,47,48].

## 2. The Immune Response in COVID-19 Related ARDS

Although the pathogenesis of COVID-19-related ARDS is multifactorial and still not completely clear, a central role of the immune response associated with SARS-CoV-2 infection has been well established [5,49,50]. Immune response in SARS-CoV-2 infection is a double-edged sword, resulting on one side as a beneficial antiviral resistance system protecting from viral infection and dissemination but on the other side as a harmful uncontrolled inflammatory system leading to tissue damage. Indeed, in severe COVID-19, the dysregulated activation of the local (respiratory tract) and systemic innate and adaptive arms of immunity results in hyper-inflammatory and fibrotic processes in the lungs, leading to ARDS and eventually progressing in shock, sepsis, and multiorgan failure [51].

Innate immunity plays a pivotal role in the pathogenesis of COVID-19-related ARDS. It represents not only the early immune response to SARS-CoV-2 infection, which, in the best of cases, eliminates SARS-CoV-2 without the contribution of the adaptive arm of the immune response [5], but also the immune response that orchestrates the subsequent adaptive immune response, determining the different clinical outcomes of COVID-19 [5,52]. Therefore, to better understand the immunopathology of ARDS, we have to focus our attention on innate immunity in the lung. This consists of mucosal pulmonary barriers composed of bronchial and alveolar epithelial cells, endothelial cells, as well as tissue-resident alveolar macrophages (sentinels of the innate response against respiratory pathogens). All these cells sense SARS-CoV-2 proteins and RNA through pattern recognition receptors (PRRs), such as Toll-like receptors (TLRs), very likely through cell membrane TLR2, endosomal TLR3, and TLR7/8, as well as by the cytosolic retinoic acid-inducible gene-1 (RIG-1) receptor [50,52,53]. These receptors trigger intracellular signaling pathways, which activate the anti-viral type I interferon (IFN) response program and the nuclear factor (NF)-κB-mediated inflammatory response, leading to cellular expression and secretion of anti-viral cytokines and inflammatory soluble mediators, such as pro-inflammatory cytokines and chemokines. Then, soluble mediators secreted by alveolar macrophages, lung epithelial cells, and endothelial cells activate and recruit peripheral blood inflammatory cells, including neutrophils and monocytes. Moreover, dendritic cell (DC) maturation is induced by SARS-CoV-2, allowing their antigen-presenting cell (APC) function and subsequent activation of T lymphocytes. In fact, innate immunity eventually activates cells of the adaptive immune response, consisting of T lymphocytes, including CD4+ T helper (Th) cell subpopulations and CD8+ cytotoxic T lymphocytes (CTLs), as well as antibody-producing B lymphocytes [50,54]. The antiviral resistance largely depends on innate type I IFN responses, associated with soluble mediators and anti-viral cytotoxic lymphocytes of the innate and adaptive immunity, such as natural killer (NK) cells and CTLs, respectively. Finally, the recovery from COVID-19 is associated with the regulation of the inflammatory response and the re-establishment of immune homeostasis in the lung and the peripheral blood.

Unlike patients who recover from COVID-19, patients undergoing COVID-19 progression in ARDS show alterations in their immune profiling concerning both innate and adaptive immune responses in the lung and the peripheral blood (Table 3) [5,55,56,57].

Alterations mainly consist of deviated innate immunity associated with dysregulated and impaired adaptive immune responses, leading to the failure in local and systemic immune regulations and homeostasis. In patients undergoing ARDS, the sensing of SARS-CoV-2 via TLRs and RIG-1 by alveolar epithelial cells, endothelial cells and alveolar macrophages results in multiple alterations of innate immune responses. Particular attention has been given to the suppressed antiviral immune response induced by the early stage of virus infection in the lungs, mainly consisting of the early inhibition of IFN-I responses, which allows high levels of virus replication in pulmonary cells [58]. Subsequently to this early phase, the excessive virus replication in patients with ARDS promotes excessive IFN response associated with excessive production of inflammatory mediators, like interleukin-(IL)-1β, IL-6, tumor necrosis factor (TNF) α and neutrophil-recruiting chemokines (e.g., CXCL8/IL-8) [55,56] by immune and non-immune pulmonary cells. Overproduction of inflammatory mediators activates endothelial cells and induces excessive recruitment in the lung of circulating inflammatory cells, mostly composed of monocytes, neutrophils, and T cells [50,59,60,61]. The immunopathology of ARDS largely depends on the phenotype and the functionality of monocytes/macrophages in the lung, including resident alveolar macrophages and pulmonary infiltrating peripheral blood-derived monocytes (Table 3) [59]. Indeed, the ratio and the balance between pro-inflammatory (M1) and anti-inflammatory (M2) macrophages strongly influence the course and severity of lung pathology [60]. Peripheral blood-derived monocytes (mainly M1 macrophages), recruited by cytokines into the alveolar space and activated by IFN-γ, secrete a further amount of pro-inflammatory cytokines and induce apoptosis of alveolar cells through the release of TNFα. Monocytes and macrophages in the lung also express high NLRP3 inflammasome (Table 3) [62], a cytosolic multiprotein complex, which facilitates the release of IL-1*β* and IL-18 cytokines, leading to inflammatory lytic cell death. Macrophages also express low levels of major histocompatibility complex (MHC) surface molecules, resulting in the reduction in their APC function, as well as the APC function of DCs is also decreased in severe acute COVID-19 (Table 3) [63,64]. Then, pulmonary macrophages acquire a profibrotic transcriptional phenotype, and the pulmonary pro-inflammatory milieu can overwhelm homeostatic tissue repair functions mediated by anti-inflammatory M2 macrophages [56,59,60], resulting in severe tissue damage, depletion of lung immune cells and pulmonary fibrosis of ARDS.

In addition, excessive infiltration and inflammatory degranulation of neutrophils contribute to permanent damage to alveolar and endothelial cells, breaking the alveolar-capillary barrier (Table 3) [52,61]. Neutrophils in ARDS also appear as myeloid-derived suppressor-like cells (MDSC-like), which may delay the clearance of SARS-CoV-2 and inhibit T cell proliferation and function [5]. Furthermore, neutrophils overexpress cellular programs necessary for neutrophil extracellular traps (NET)-formation (Table 3). Indeed, NET has a central role in ARDS because it traps inflammatory cells, preventing the recruitment of tissue-repairing cells, as well as driving vascular occlusion, which is particularly dangerous in the microvasculature, leading to severe organ damage [61].

Therefore, excessive pulmonary infiltration of inflammatory monocytes and neutrophils further raises pro-inflammatory cytokines and chemokine levels in the lung (e.g., TNFα, IL-6, IL-1β, and CXCL8/IL-8) and in the bronchoalveolar lavage fluid (BALF) (e.g., CCL2/MCP-1, CCL3/MIP-1α, CCL4/MIP-1β, and CXCL10/IP-10), promoting alveolar epithelial cell death, hyperactivation of signal transducer and activated transcriptions (STATs) signaling pathways, leading thus to serious local and systemic dysregulation of immune homeostasis (Table 3). Hence, the inflammatory environment in the lung of patients with COVID-19-related ARDS is also associated with elevated levels of inflammatory cytokines and chemokines in the peripheral blood (e.g., IL-1β, TNFα, IL-6, G-CSF, GM-CSF, IL-1RA, IFN-γ, IL-17, CCL2/MCP-1, CCL3/MIP-1a, CCL5/RANTES, CCL8/MCP-2, CXCL2/MIP-2, CXCL8/IL-8, CXCL9/MIG, CXCL10/IP-10 and CXCL16) [49,52,55], associated to increased levels of anti-inflammatory cytokines, such as IL-10 and TGF-β [57]. Noteworthy, these anti-inflammatory cytokines can suppress circulating leukocyte function; in particular, TGF-β may trigger a process of pulmonary fibrosis, while the regeneration of lung epithelia is impaired [57].

Then, a massive increase of local and circulating pro-inflammatory cytokines, such as IL-6, is accompanied by elevated lung and circulating levels of humoral innate immunity pattern recognition molecules, including C-reactive protein (CRP), mannose-binding lectin (MBL), long pentraxin PTX3, and complement (Table 3) [65,66,67], whose activation further contributes to lung inflammation and damage in ARDS. In fact, SARS-CoV-2 infection of alveolar epithelial cells and endothelial cells may trigger complement hyperactivation via the three complement activation pathways. Deposit of complement membrane attack complex (MAC) on the membranes of alveolar epithelial and endothelial cells leads to cell lysis, while the deposit of complement components in pulmonary microvessels may promote hypercoagulability and thrombosis [67].

Blood hallmarks of severe COVID-19 have been established as increased neutrophils (neutrophilia) and decreased lymphocytes (lymphopenia) (Table 3). Indeed, a decreased number of anti-viral lymphocytes of innate and adaptive immunity, such as NK cells and CTLs, respectively, have been observed in severe COVID-19 (Table 3) [5,68]. Lymphopenia also includes other cells of the adaptive arm of the immune response, where a marked decrease of circulating CD4+ and B lymphocytes, also associated with evidence of lymphocyte dysfunction, was reported in ARDS [54]. Indeed, NK cells and CTLs appeared to be exhausted with a reduced ability to produce anti-virus defense molecules, such as CD107a, IFN-γ, IL-2, granzyme B, and TNFα [68]. CTLs from patients with ARDS also have significantly higher expression of the inhibitory molecule programmed death (PD)-1 and T-cell immunoglobulin and mucin domain 3 (Tim-3), further indicative of T-cell exhaustion. Another event associated with COVID-19-related ARDS is the imbalance between Th17 and Treg lymphocytes (Table 3) [50]. An increased number of Th17 cells plays an important role in COVID-19 progression in ARDS, by promoting tissue neutrophil recruitment and activating the cytokine cascade (through secretion of IL-17). A decreased number and function of Treg cells impaired regulation of inflammation and immune response homeostasis, contributing thus to the uncontrolled inflammatory status in ARDS [50]. Concerning B cells in ARDS, defects in B cell differentiation due to the loss of germinal center lead to late antibody response and production of low affinity and low neutralizing antibodies (Table 3) [50].

**Table 3:** Immunological biomarkers associated with COVID-19 related ARDS.

Innate Immunity in the Lung	Dysregulation and References
Alveolar epithelial cells	type I IFN response and IFN-stimulated genes [5,58] NF-κB-dependent pro-inflammatory mediators [51,55,56]: − cytokines: IL-6, TNFα, IL-1β − chemokines: CCL20, CXCL1, 2/MIP-2, 3/MIP-1a [5,6,8,16] STAT signaling [51] NLRP3 inflammasome [62]
Resident alveolar macrophages and monocytes/macrophages	NF-κB-dependent pro-inflammatory mediators [51,55,56]: − cytokines: IL-6, TNFα, IL-1β, CXCL8/IL-8 − chemokines: CXCL10/IP-10 STAT signaling [51] NLRP3 inflammasome [5,56] M1/M2 balance [59,60] Profibrotic transcriptional phenotype [5,60] APC function [5,59]
Neutrophils	number (neutrophilia) [5,52] activation and degranulation [61] MDSC-like phenotype [5] NET formation [5,61]
DCs	APC function [5,63,64]
NK cells	number (lymphopenia) [5] function (exhausted phenotype) [68]
Humoral pattern recognition molecules	CRP [51,66] PTX3 long pentraxin [5,51,65,66] MBL [5,51,66] Complement [5,50,51,67]
**Adaptive immunity in the lung**	**Dysregulation**
T lymphocytes	number (lymphopenia: Th cells, CTLs) [5,50,54] function (exhausted phenotype) [50,68] Th17/Treg balance [50]
B lymphocytes	differentiation and function [5,54] antibody response [50] production of affinity and neutralizing anti bodies [50]

APC: antigen presenting cell; CCL: C–C motif chemokine ligand; CTLs: cytotoxic T lymphocytes; CRP: C-reactive protein; CXCL: C–X–C motif chemokine ligand; DCs: dendritic cells; IFN: interferon; IL-1β: interleukin-1β; IL-6: interleukin-6; IP-10: IFN-γ-inducible protein 10; MBL: mannose-binding lectin; MDSC: Myeloid-derived suppressor cells; MIP: Macrophage Inflammatory Protein; NET: Neutrophil extracellular trap; NF-κB: nuclear factor-κB; NK: natural killer; NLRP3: NOD-, LRR- and pyrin domain-containing protein 3; PTX3: pentraxin 3; STAT: signal transducer and activator of transcription; TNFα: tumor necrosis factorα; Treg: regulatory T cells.

In conclusion, accumulating evidence suggests that ARDS results from highly inflammatory and dysfunctional immune responses involving both the innate and the adaptive arms of the immune system, although a major role for the innate immune response in promoting the dysregulated immune responses leading to ARDS is largely emphasized.

## 3. n-3 PUFAs and the Immunomodulatory Activity Relevant to COVID-19 Related ARDS

**Figure 1:**
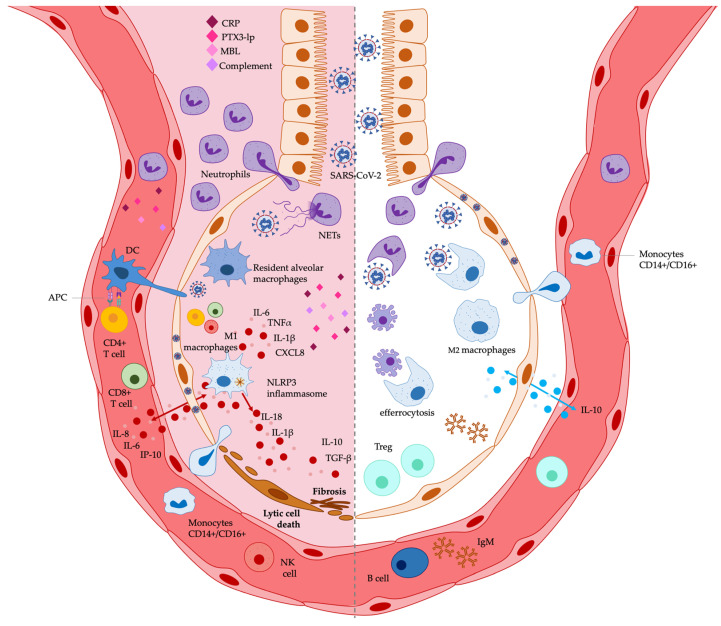
Potential effects exerted by n-3 PUFAs on lung immunological markers associated with COVID-19 related ARDS. The immunological biomarkers potentially decreased by n-3 PUFAs are shown on the left, while those potentially increased are shown on the right. See Table 4 and the text for a detailed description. APC: antigen presenting cell; CRP: C-reactive protein; DCs: dendritic cells; IgM: immunoglobulin M; IL-: interleukin-; IP-10: IFN-γ-inducible protein 10; MBL: mannose-binding lectin; NET: neutrophil extracellular trap; NK: natural killer; NLRP3: NOD-, LRR- and pyrin domain-containing protein 3; PTX3-lp: long-pentraxin 3; Treg: regulatory T cells.

A plethora of studies has reported the immunomodulatory activity of EPA and DHA in vitro and in vivo [11,12,13,14,15,16,17,18]. n-3 PUFAs can cause multiple effects on innate and adaptive immune responses, including the regulation of immune and non-immune cell functions, mainly by inhibiting cell expression of pro-inflammatory cytokines and chemokines, as well as by the generation of SPMs, involved in the resolution of inflammation. Several studies have also shed light on the specific mechanisms of action underlying the immunomodulation by n-3 PUFAs, mainly highlighting their capability of being incorporated in the cell surface membrane, regulating thus the function of surface receptors, including TLRs, as well as signaling pathways, such as the NF-κB-mediated inflammatory pathway. A detailed spectrum of the immunomodulatory activities exerted by n-3 PUFAs is reviewed elsewhere [11,12,13,14,15,16,17,18]. In this review, we focus our attention on DHA and EPA immunomodulatory activities on the immune environment present in the lung and relevant to inflammatory stimuli leading to ARDS, preferentially viral infections, or experimental models relevant to SARS-CoV-2 infection. Therefore, we reference n-3 PUFAs immunomodulation in pulmonary and ARDS experimental models performed in vitro (in cell cultures) and in vivo (in pre-clinical and clinical studies). We have summarized these activities in Table 4 and Figure 1, focusing on the immunomodulation by PUFAs of immune cell functions and immune soluble factors considered as immunological biomarkers associated with COVID-19-related ARDS (Table 3).

### 3.1. PUFAs and ARDS: In Vitro Immunomodulatory Effects on Lung Epithelial Cells and Macrophages

Very few investigations have analyzed the *in vitro* immunomodulatory activity of n-3 PUFAs on airway epithelial cell models or airway-infected cell experimental models relevant to SARS-CoV-2 human infection. In 2009, Saedisomeolia et al. suggested a potential role of DHA in suppressing rhinovirus-induced airway inflammation. They measured the release of IL-6, IL-8, and IFN-γ-induced protein-10 (IP-10) by Calu-3 human lung epithelial cells, pre-incubated with EPA, DHA and arachidonic acid (AA) for 24 h, and then infected with rhinovirus for 48 h. They found that pre-treatment with EPA and AA did not change the release of inflammatory biomarkers, whereas pre-treatment with DHA (400 mM) significantly reduced the release of IL-6 and IP-10 from infected cells, and the cellular DHA content negatively correlated with cytokine release. However, no effect was observed on IL-8 production. In addition, none of the n-3 PUFAs significantly modified rhinovirus replication [69]. Then, Cotogni et al. reported that the release of pro-inflammatory molecules by LPS-stimulated A549 human alveolar epithelial cells depends on the n-3(DHA)/n-6(AA) PUFA ratio in cell membranes. Indeed, the supply of 1:1 and 1:2 DHA/AA ratios, significantly inhibited the release of TNFα, IL-6, and IL-8 inflammatory cytokines, whereas 1:4 and 1:7 DHA/AA ratios, increased their release. The 1:1 and 1:2 ratios also increased the release of the IL-10 anti-inflammatory cytokine [70]. Then, accordingly, the 1:2 DHA/AA ratio reduced the inflammatory response by A549 human alveolar cells exposed to an ex vivo inflammatory stimulus, such as the BALF collected from patients with ARDS (12 patients: 7 with pneumonia and 5 with sepsis). The investigators exposed A549 cells to the BALF, and after 18 h, DHA and AA were added in 1:2 or 1:7 ratios; 24 h later, the inflammatory response was evaluated. TNFα, IL-6, and IL-8 pro-inflammatory cytokine release was reduced by the 1:2 ratio, whereas it was increased by the 1:7 ratio. The 1:2 ratio also reduced COX-2 cell content and PGE2 release, as well as NF-κB translocation into the nucleus, while it increased PPARγ activation and IL-10 release [71]. Although these few studies indicate the potential anti-inflammatory activity of DHA on lung epithelial cells, to the best of our knowledge, no study exists investigating whether n-3 PUFAs affect inflammatory responses *in vitro* by using airway epithelial cell models relevant to human infection by SARS-CoV-2, such as Calu-3 cells, a human lung-derived epithelial cell line that is permissive to SARS-CoV-2 infection, or primary bronchial cells, such as a three-dimensional (3D) human bronchial epithelial cell (HBEC) model, following SARS-Co-V2 infection [66].

Extensive investigation highlights the capability of n-3 PUFAs to regulate the functions of monocytes and macrophages (Table 4), resulting these innate immune cells as the major cell type responsible for the modulation of inflammation by n-3 PUFAs [11]. Downregulation of LPS-induced cytokine gene expression by DHA or EPA is demonstrated in several studies using different monocyte/macrophage experimental models, including macrophage cell lines (such as murine Raw264.7 or human THP-1 cells), as well as murine primary macrophages (such as bone marrow- and peritoneal-derived macrophages) [72,73,74,75,76,77,78]. Looking at the experimental designs of the different studies, it should be noted that although the timing of the administration of n-3 PUFAs, with respect to the inflammation, seems to play an important role in the efficacy of their immunomodulatory effects, most of the experiments were performed using PUFA treatment before the inflammatory stimulus. Indeed, DHA pre-treatment of LPS-stimulated human primary monocyte-derived macrophages decreased (25%) 579 inflammation-related mRNA transcripts [77]. Moreover, EPA or DHA pre-treatment of primary macrophages and macrophage cell lines decreased LPS-induced phosphorylation, nuclear translocation, and transcriptional activity of NF-κB (Table 4) [72]. Furthermore, EPA or DHA pre-treatment also inhibited LPS-induced activation of other transcription factors such as IRF3, STAT-1, STAT-3, IRF1, ERK1/2, JNK, and MAPK (Table 4) [77]. On the other hand, Allam-Ndoul et al. have reported that in THP-1-derived macrophages, the most effective downregulation of LPS-induced mRNA expression of inflammatory genes (including those for IL-6, TNF-α, IL-1β, and MCP1) was achieved after LPS stimulation for EPA treatment, but during co-incubation with LPS for DHA [78]. In addition, some studies performed in murine or human macrophages reported additive immunomodulatory effects by co-incubating EPA and DHA together [11,78]. We should point out that the anti-inflammatory properties of EPA and DHA on macrophages were not peculiar to bacterial LPS-induced inflammatory stimulus. Indeed, DHA and EPA also decreased pro-inflammatory cytokine secretion by Raw264.7 macrophages infected with other extracellular bacteria, such as *Rhodococcus equi* or *Pseudomonia aeruginosa*, while increased the secretion of the IL-10 anti-inflammatory cytokine (Table 4) [79]. The capability of n-3 PUFAs to inhibit inflammatory cytokine production by macrophages is also due to their potent suppression activity on inflammasome-mediated inflammation (Table 4). In fact, EPA and DHA inhibited the NLRP3 inflammasome in macrophage cell lines as well as in primary human and murine macrophages [74,80]. In addition, the capability of n-3 PUFAs to inhibit the pro-inflammatory cytokine production by macrophages upon LPS stimulation is also due to their capability of downregulating M1 macrophage polarization, while promoting M2 polarization in macrophage cell lines and mouse primary macrophages [73]. However, although n-3 PUFAs decrease macrophage M1 polarization they can increase the phagocytic function of macrophages, as reported for the engulfment of extracellular bacteria, such as *Rhodococcus equi*, *Pseudomonia aeruginosa* and *Escherichia coli* (Table 4) [81,82]. Finally, PUFAs can modulate the APC macrophage function, thus affecting the activation of adaptive immunity (i.e., T lymphocytes). In fact, *in vitro*, and in vivo studies also indicate that MHC class I and class II molecule expressions and antigen presentation to T cells are reduced in APCs, such as macrophages and DCs, exposed to EPA or DHA [83,84]. These multiple studies indicate the capability of n-3 PUFAs to inhibit the expression and the secretion of pro-inflammatory cytokines and chemokines by macrophages while stimulating their phagocytic function. They also report the inhibition by n-3 PUFAs of macrophage and DC APC function towards T lymphocytes, thus impairing T lymphocyte activation.

Therefore, by analyzing the above-mentioned data and considering the proposal to use n-3 PUFAs as potential immunomodulatory agents in COVID-19 and COVID-19-related ARDS, we have to make two considerations. The first consideration is that all these studies have been designed using extracellular bacterial pathogens (bacterial LPS, *Rhodococcus equi*, *Pseudomonas aeruginosa*, *Escherichia coli*) as experimental inflammatory stimuli, and, to the best of our knowledge, no study exists investigating whether n-3 PUFAs can affect the *in vitro* inflammatory responses of macrophages stimulated by intracellular pathogens such as viruses. This is an important point since if it is true that PUFAs act on pathogen receptor expression and function, the receptors sensing extracellular pathogens are different from those sensing intracellular viral pathogens. This might imply that the immunomodulatory activity of PUFAs on macrophages stimulated by viruses might be different from that observed following extracellular bacterial stimulation. In fact, inflammation induced by bacterial LPS is triggered by the interaction between LPS and TLR4 receptor, whose signaling pathway induces the activation of transcription factors, such as NF-κB, MAPK, or ERK, leading to downstream gene expression of inflammatory cytokines and chemokines [85]. Differently, viruses, including the SARS-CoV-2 RNA virus, are sensed by surface TLR2, endosomal TLR3, TLR7, and TLR8 or cytoplasmic RIG-1 like receptors, which lead to the activation of NF-κB and IRFs, leading to inflammatory cytokine production and type I IFN production, respectively [5,52,53]. Therefore, there is a need to investigate the effects of PUFAs on *in vitro* macrophage functions in experimental virus models relevant to SARS-CoV-2 human infections. The second consideration is that the *in vitro* results have been mostly obtained by using macrophage cell lines or primary bone marrow- or peritoneal-derived macrophages, and not macrophages relevant to respiratory viral infections. Therefore, there is a need to analyze n-3 PUFA activities on pulmonary macrophages, such as mice-derived primary alveolar macrophages [86].

### 3.2. PUFAs and ARDS: Immunomodulation in Animal Lung Experimental Models

Various experimental pre-clinical in vivo models of pneumonia, acute lung injury (ALI) or ARDS investigate n-3 PUFA supplementation for the modulation of inflammation-derived pulmonary tissue damage mediated by the innate and the adaptive arm of the immune response. Mancuso et al. suggested a beneficial effect of n-3 PUFAs over n-6 PUFAs in ALI caused by *Salmonella* endotoxin intravenous injection. Indeed, they observed decreased severity of pulmonary microvascular protein permeability and decreased pulmonary infiltration of neutrophils in rats fed enteral n-3 PUFAs enriched diet (21 days), compared to rats fed n-6 PUFAs. In addition, alveolar macrophages expressed lower concentrations of n-6(AA)-derived pro-inflammatory metabolites [87,88]. Then, Sharma et al. indicated that n-3 PUFA dietary supplementation in mice could exert a beneficial effect against acute pneumonia caused by *Klebsiella pneumoniae*. In fact, although no effect on the establishment of the infection was observed after two weeks of n-3 PUFA feeding, an improved resistance, as reduced lung bacterial load associated with improvement in pathology, was observed after six weeks of n-3 PUFA administration. Lower lung levels of nitric oxide, malondialdehyde and lactate dehydrogenase were associated with decreased severity of tissue damage. Interestingly, there was also a significant increase in the lung levels of TNFα and IL-1β pro-inflammatory cytokines, while no significant change was observed in IL-10 levels. Moreover, alveolar macrophages exhibited a significant decrease in the level of apoptosis and enhanced *in vitro* phagocytic activity [89]. Furthermore, it has been reported that n-3 PUFA-derived SPMs have beneficial effects on pulmonary inflammation [90,91], and their beneficial role has been investigated in different ALI or ARDS experimental models. Indeed, in mice with cigarette smoke-induced ALI, the administration of resolving (Rv)D1, concurrently with cigarette smoke exposure, significantly reduced neutrophilic lung inflammation and production of pro-inflammatory cytokines (Table 4), while increasing the anti-inflammatory cytokine IL-10 and promoted differentiation of M2 macrophages and neutrophil efferocytosis. Moreover, the administration of RvD1 after the final smoke exposure accelerated the resolution of lung inflammation [92]. Accordingly, RvE1 (approximately 0.005 mg/kg) intravenous (i.v.) infusion in ALI, caused by lung acid aspiration (hydrochloric acid) and subsequent bacterial (*Escherichia coli*) challenge, inhibited neutrophil-mediated inflammation while preserving host defense. RvE1 significantly decreased lung tissue levels of several pro-inflammatory chemokines and cytokines, including IL-1 β, IL-6, HMGB-1, MIP-1a, MIP-1b, keratinocyte-derived chemokine, and MCP-1, in a manner independent of the anti-inflammatory mediators IL-10 and lipoxin A4. Furthermore, RvE1-treated animals had a marked improvement in survival [93]. In addition, a certain number of studies also have suggested that SPMs can decrease the severity and duration of ARDS by improving alveolar fluid clearance and decreasing excessive inflammation. For example, in mice with LPS-induced ALI, RvD1 treatment helped to protect mice and improve lung pathology; it reduced TNFα and pulmonary neutrophil infiltration [94]. Additionally, protectin DX improved lung histopathology, reduced lung inflammation and mitigated pulmonary edema in a mouse model of LPS-induced ALI [95]. Very recently, encouraging results have also been reported by the delivery of n-3 PUFAs via inhalation in LPS-induced ALI in rats. Nebulized treatment promoted the decrease of alveolar histiocytosis severity, IL-6, TNFα, and IL-1β, as well as TGF-β and IL-10 levels [96].

Although the above-mentioned pre-clinical studies indicate beneficial immunomodulatory effects by n-3 PUFAs on animal pulmonary inflammatory conditions, as for in vitro studies, most of these in vivo investigations used extracellular bacterial infections as lung inflammatory models, whereas it should be noted that contradictory results have been obtained in lung inflammation due to intracellular pathogens, including intracellular bacterial and viral lung infections. In fact, a study aimed to determine the role of dietary n-3 PUFAs and n-6 PUFAs on immunity and resistance to aerosol infection with virulent *Mycobacterium tuberculosis* in guinea pigs’ diets, documented the adverse immunomodulatory effects of n-3 PUFA consumption in the context of tuberculosis resistance. Dietary n-3 PUFA consumption reduced in vivo skin test and *in vitro* lymphoproliferative responses relative to n-6 PUFAs consumption, reflecting the loss of antigen-specific T-cell functions. In addition, at 3- and 6-week post-infection, n-3 PUFA-fed guinea pigs had more viable mycobacteria in the lungs compared with n-6 PUFA-fed guinea pigs [97]. Then, concerning lung inflammatory experimental models using viral infections, Byleved et al. reported a delay in influenza virus clearance and an impaired immune response in mice fed n-3 PUFAs (3 g/100 g sunflower oil with 17 g/100 g fish oil) for 14 days following intranasal challenge with live influenza virus. At day one and day five after infection, PUFA-fed mice had higher lung viral load and lower body weight than the control; they also had impaired production of lung IFN-γ, lung IgA-specific antibodies, and serum IgG, although lung IFN-α/β and the relative proportions of bronchial lymph node CD4+ and CD8+ T lymphocytes did not differ from control after infection. However, differences observed during the course of infection did not affect the ultimate outcome, as both groups (treated and control) cleared the virus by day seven [98]. In another influenza model, Schwerbroch et al. indicated that the immunomodulatory and anti-inflammatory properties of fish oil feeding negatively dampened the immune response to influenza virus infection, resulting in increased morbidity and mortality. Mice fed either a menhaden fish oil/corn oil diet (4 g fish oil:1 g corn oil, wt:wt at 5 g/100 g diet) or a control corn oil diet for two weeks were infected with influenza A/PuertoRico/8/34 and analyzed for lung pathology and immune function. Although fish oil-fed mice had lower lung inflammation compared with controls, fish oil feeding also resulted in a 40% higher mortality rate, a 70% higher lung viral load at day seven post-infection, and a prolonged recovery period following infection. Although splenic NK cell activity was suppressed in fish oil-fed mice, lung NK activity was not affected. Additionally, the lungs of infected fish oil-fed mice had significantly fewer CD8+T cells and decreased mRNA expression of macrophage IP-1-a, TNFα, and IL-6 [99]. According to these results, Kang et al. reported that n-3 PUFAs weakened the antiviral response against an acute viral infection induced by lymphocytic choriomeningitis virus (LCMV) by CD8+ T cells, potentially modulating cytotoxic and inflammatory molecule release. They observed a significant reduction of the CD8+CTL-mediated anti-viral responses in FAT-1 transgenic mice, capable of synthesizing n-3 PUFAs from n-6 PUFAs. Interestingly, the expansion of adoptively transferred wild-type (WT) LCMV-specific T cell receptor (TCR) transgenic CD8+(P14)T cells into FAT-1 mice was significantly decreased. Moreover, activation of anti-viral CD4+helper T cells was reduced in FAT-1 mice. Importantly, P14 cells carrying the fat-1 gene that were adoptively transferred into WT mice exhibited a substantially decreased ability to proliferate and produce cytokines against LCMV infection [100]. It has been also reported that a diet rich in n-3 PUFAs did not substantially affect responses to poxviral infection, an acute infection that begins in the respiratory tract and spreads by viremia to internal organs. In this experimental work, mice were fed for three weeks prior to infection and continuing during infection and recovery one of the following: 1) a normal low-fat (13% kcal) diet, 2) a low-fat diet containing n-3 PUFAs, 3) a high-fat (41% kcal) diet rich in n-3 PUFAs, 4) a high-fat n-6 PUFA diet, or 5) a high fat monounsaturated diet. The authors found no statistically significant differences in the susceptibility of mice to viral infection, morbidity, viral organ titers, recovery time, or mortality with these diets [101]. However, in contrast to the absence of effects or negative effects by n-3 PUFAs reported by many authors in lung viral infection diseases, some beneficial activity might be derived from n-3 PUFA anti-inflammatory pro-resolving metabolites such as SPMs. Indeed, Ramon et al. reported the ability of a DHA-derived SPM, SPM 17-HDHA, to enhance the adaptive immune response, promoting B lymphocyte activities against the influenza virus (H1N1). Mice immunized with H1N1-derived hemagglutinin (HA) protein plus 17-HDHA increased antigen-specific antibody titers compared to the control. In addition, 17-HDHA increased the number of antibody-secreting cells *in vitro* and the number of HA-specific antibody-secreting cells present in the bone marrow. Importantly, the increased antibody production mediated by 17-HDHA was more protective against live H1N1 influenza infection in mice [102].

The analysis of all the above-mentioned data obtained in pre-clinical experimental models in vivo shows that n-3 PUFA administration may induce both beneficial and deleterious effects in the control of infectious diseases. Immunomodulation by n-3 PUFAs mainly results in anti-inflammatory effects (Table 4) that, in some inflammatory lung conditions, can ameliorate the outcome of pulmonary disease by decreasing tissue damage due to an excessive inflammatory response. However, immunomodulation by n-3 PUFAs can also result in immunoregulatory and immunosuppressive effects (Table 4) that might impair the immune resistance against the pathogen, potentially increasing the susceptibility to pathogen infection and dissemination, resulting thus in increased morbidity and mortality [97,98,99,100,101,103]. For example, the cytotoxic activity mediated by cytotoxic lymphocytes such as NK and CD8+ CTLs against virus-infected cells is very important in fighting and controlling viral infections. Therefore, the potential capability of n-3 PUFAs to suppress anti-viral NK and CTL effector cells may be detrimental to the defense against viral pathogens. Therefore, further pre-clinical trials are needed to verify the effects of the administration of n-3 PUFAs in inflammatory lung diseases due to viral infections.

### 3.3. PUFAs and ARDS: Immunomodulation in Human Clinical Trials for Lung Diseases

Clinical trials assessing immunomodulation by n-3 PUFAs administered alone in pulmonary inflammatory diseases such as human ALI, ARDS and sepsis are limited and also provided contradictory results. Moreover, in most of the studies, n-3 PUFAs were administered in different concentrations together with other immunomodulatory agents (e.g., γ-linolenic acid-GLA and antioxidants), where the specific activity of n-3 PUFAs, as regards to the other components, is difficult to identify [104,105,106,107,108,109]. Hosny et al. reported that a short-term (7 days) high-dose EPA and DHA (9 g/d added to 1 g/d ascorbic acid, 400UI/12 h α-tocopherol and 100 μg/d selenium) diet in 37 patients with early-stage sepsis decreased the levels of inflammatory markers such as CRP, IL-6, and procalcitonin, as well as reduced the need for mechanical ventilation and the development of sepsis [110]. A meta-analysis of 25 randomized controlled trials (RCTs) studying the effects of n-3 PUFA enteral nutrition on sepsis (including ARDS-induced sepsis) found support for an effect on mortality, although a paucity of high-quality evidence led to limited conclusions [111]. Recently, Dirjaianto et al., analyzing a literature review yielding 12 studies (6474 subjects) on the efficacy of n-3 PUFAs as a potential adjunct treatment in slowing down chronic obstructive pulmonary disease (COPD) progression, report that, although n-3 PUFAs led to a reduction in inflammation, the association for lung function was weak [112]. Of note, Stapleton et al. have designed a phase II double-blind placebo-controlled RCT to determine the efficacy of enteral n-3 PUFAs alone in reducing pulmonary and systemic inflammation in patients with ALI (41 patients received n-3 PUFAs and 49 received placebo). Although enteral n-3 PUFA feeding was absorbed well and was safe in critically ill patients, the authors did not find a decrease in any pulmonary (BALF) or systemic (plasma) inflammatory biomarker (including their primary endpoint IL-8), and they did not find any differences in organ failure, ventilator-free days, ICU-free days, or mortality [113]. Recently, Dushianthan et al., in a Cochrane meta-analysis of 10 studies (1015 participants), noted a statistical reduction in mortality from ARDS when n-3 PUFAs were compared with a lipid-rich enteral formula, but they also stated that it was uncertain whether n-3 PUFA supplementation altered mortality, oxygenation, or duration of mechanical ventilation and intensive care unit (ICU) stays, because of large heterogeneity between the studies [114]. Therefore, there are many uncertainties and divergences on the potential positive effects, in terms of immune and inflammatory responses as well as respiratory and clinical outcomes, for enteral n-3 PUFAs supplementation in clinical trials for lung diseases. Then, the administration of n-3 PUFAs by i.v. infusion has been proposed as a more effective strategy to increase their potential immunomodulatory activity in the setting of severe acute inflammatory diseases and intensive care, including COVID-19-related ARDS [115,116]. In a study of nineteen patients in septic shock, ten patients randomized to i.v. n-3 PUFA emulsion (350 mL/day, equivalent to 14 g DHA and EPA) for three days attained an n-3/n-6 ratio of 2.5:1 and remarkably lowered levels of TNF-α, IL-6, and IL-8 in ex vivo leukocytes stimulated with endotoxin from *Salmonella typhimurium* [117]. Then, in an RCT performed to evaluate the effects of i.v. n-3 PUFA emulsion (0.1 g/kg/day for 14 days) in ventilated patients with ARDS (n = 61), it was reported that, although i.v. n-3 PUFAs alone did not improve ventilation, length of ICU stay, or survival, the observed fall in PaO2/FiO2 ratio from baseline to day 14 was significantly higher in the control group as compared to the patients (=31) treated with i.v. n-3 PUFA emulsion [118]. Hence, according to the results obtained for enteral n-3 PUFA supplementation, evidence from clinical trials using i.v. n-PUFA infusion is insufficient to determine whether this route of administration offers a potential advantage in a clinical setting that would be relevant to COVID-19 and COVID-19-related ARDS. Therefore, clinical evidence from trials assessing the role of n-3 PUFAs in ameliorating ARDS is limited and with small samples. Therefore, future larger randomized, blinded clinical trials are warranted, and sufficient confirmatory results are required to further shed light on this topic.

**Table 4:** PUFAs effects on Immunological Biomarkers associated with COVID-19 related ARDS.

Target: Innate Immune Cells and Molecules	Effects and References
Alveolar epithelial cells	↓ NF-κB activation [70] ↓ cytokine production: IL-6, IL-8, IP-10 [69,70,71,94,97] ↑ cytokines production: IL-10 [97]
Monocytes/Macrophages	↓ NF-κB activation [27,72,80] ↓ STAT, IRF, JNK, MAPK signaling [11,77] ↓ cytokine production [11,78,99] ↓ chemokine production [11,94] ↓ NLRP3 inflammasome [74,80] ↓ M1 polarization ↑ M2 polarization [11,73,90,91] ↑ phagocytosis and efferocytosis [11,81,82,86,89] ↓ APC function [11,84]
Neutrophils	↓ migration and tissue infiltration [11,87,88,94] ↑ phagocytosis [11,90,91] ↓ NET formation [11]
DCs	↓ APC function [11,83,84]
NK cells	↓ anti-viral function [11]
Humoral pattern recognition molecules	↓ CRP [20,110] ↓ PTX3 long pentraxin [119] ↓ MBL [120] ↓ Complement [121]
**Target: adaptive immune cells**	**Effects and References**
T lymphocytes	↓CTL (CD8+) and T helper (CD4+) [99,100] ↓ activation and function [11,97,100] ↑Treg [11,12]
B lymphocytes	↑ ↓ activation * [11,27,98,102] ↑ IgM production [11]

* The double arrows indicate literature discordance. Up arrow: increase in molecules or activities. Down arrow: decrease in molecules or activities. APC: antigen presenting cell; CRP: C-reactive protein; CTL: cytotoxic T lymphocytes; DCs: dendritic cells; IgM: immunoglobulin M; IL-: interleukin-; IP-10: IFN-γ-inducible protein 10; IRF: Interferon regulatory factors; JNK: c-Jun N-terminal kinase; MAPK: mitogen-activated protein kinase; MBL: mannose-binding lectin; NET: neutrophil extracellular trap; NF-κB: nuclear factor-κB; NK: natural killer; NLRP3: NOD-, LRR- and pyrin domain-containing protein 3; PTX3: pentraxin 3; STAT: signal transducer and activator of transcription; Treg: regulatory T cells.

## 4. Conclusions

The analysis of the above-mentioned studies on the therapeutic potential of n-3 PUFA intake in the prevention and/or control of severe inflammatory lung diseases such as ARDS, shows that, despite some encouraging data, there are controversial results in terms of the potential positive effects on immune and inflammatory markers as well as on respiratory and clinical outcomes; indeed, there is still limited evidence on n-3 PUFA effectiveness. Therefore, to establish the therapeutic value of n-3 PUFAs in COVID-19-related ARDS, we think there is a need for additional investigation on the immunoregulatory effects of n-3 PUFAs in pre-clinical viral experimental models relevant to SARS-CoV-2 human infection as well as the need for further larger randomized clinical trials. We assume that the effectiveness of n-3 PUFA therapeutic intake might depend not only on the specific type of pathogen causing ARDS but also on the dose and timing of n-3 PUFA administration. Concerning this last point, we consider that the potential therapeutic efficacy of immunomodulation by n-3 PUFAs might also depend on the time of n-3 PUFA administration with respect to the inflammatory stimulus and the inflammatory response phase underlying ARDS immunopathology.

## Figures and Tables

**Table 1:** Clinical trials approved for the use of n-3 PUFAs for COVID-19 prevention.

Study Identifier and References	Intervention(s)	Title of the Study and Proposal	Recruitment Status
NCT04505098 Kaiser Permanente Northern California (KPNC). Phase 4 [36]	Icosapent ethyl (IPE)/Vascepa 2 g by mouth twice daily for at least 6 months.	MITIGATE: A Pragmatic Randomized Trial of Icosapent Ethyl for High-Cardiovascular Risk Adults. A prospective, open-label, parallel-group, randomized, pragmatic clinical trial designed to evaluate the real-world clinical effectiveness of pre-treatment with IPE/Vascepa^®^, compared to the usual standard of care to prevent and reduce the sequelae of laboratory-confirmed viral upper respiratory infection (URI)-related (i.e., COVID-19, influenza, and other known viral respiratory pathogens) morbidity and mortality in a high-risk cohort of adults with established atherosclerotic cardiovascular disease.	Active, not recruiting. Estimated enrollment: 39,600 participants, 50 years and older, with no prior history of confirmed COVID-19.
NCT04460651 Estudios Clínicos Latino América. Phase 3 [37]	Icosapent ethyl (IPE)/Vascepa 8 g (4 capsules every 12 h) for the first 3 days, followed by 4 g (2 capsules every 12 h) thereafter for 4–60 days.	PREPARE-IT: Prevention of COVID-19 With EPA in Subjects at Risk Intervention Trial. The trial intended to reduce infection rates and subsequent morbidity and mortality among subjects at high risk of COVID-19 infection.	Completed. Enrollment: 18 years and older, any subject that is circulating and exposed to the public.
NCT04483271 Mahmoud Suleiman Abu-Samak; Applied Science Private University, Jordan. (Not applicable phase) [38]	Dietary Supplement: 300 mg of omega-3 fatty acid/daily for 2 months.	The Effect of Omega-3 Supplements on the Serum Levels of Selected Cytokines (IL-1, IL-6, TNF) Involved in Cytokine Storm of Covid-19. A randomized clinical trial designed to evaluate the effect of daily 300 mg of omega-3 fatty acid supplements on the immune health status of uninfected people with Covid-19 as a part of preventive health care.	Enrolling. Estimated enrollment: 100 participants in the age range of 30 to 66 years without a medical diagnosis of COVID-19 infection.

EPA: eicosapentaenoic acid; i.v.: intravenous; ICU: intensive care unit; IL-1: interleukin-1; IL-6: interleukin-6; IPE: icosapent ethyl; PO/NGT/OGT: PO = *per os*/by mouth/NGT = nasogastric tube/OGT = orogastric tube; TNF: tumor necrosis factor; URI: upper respiratory infection.

**Table 2:** Clinical trials approved for the use of n-3 PUFAs for COVID-19 cure.

Study Identifier and References	Intervention(s)	Title of the Study and Proposal	Recruitment Status
NCT04647604 Karolinska University Hospital. Phase 2 [39]	Omegaven^®^ (2 mL/kg/day, equivalent to 6 g DHA+EPA in a 70 kg individual) once daily i.v. for 5 days; 7 patients/each group received concomitant corticoids	Resolving inflammatory storm in COVID-19 patients by Omega-3 Polyunsaturated Fatty Acids.	Completed. 22 older participants with COVID-19 diagnosis and requiring hospitalization.
NCT04412018 Canadian Medical and Surgical Knowledge Translation Research Group. Phase 2 [40]	Icosapent ethyl (IPE)/Vascepa: 4 g by mouth twice daily for 3 days; then 2 g twice daily for the subsequent 11 days	VASCEPA-COVID-19: An Investigation on the Effects of Icosapent Ethyl (VascepaTM) on Inflammatory Biomarkers in Individuals With COVID-19.	Completed. 100 participants
NCT04460651 Estudios Clínicos Latino América, Argentina. Phase 3 [37]	Icosapent ethyl (IPE)/Vascepa 8 g (4 capsules every 12 h) for the first 3 days; followed by 4 g (2 capsules every 12 h) thereafter days 4–28.	PREPARE-IT: Treatment of COVID-19 With EPA in Subjects at Risk—Intervention Trial. The PREPARE-IT investigator-initiated trial program is a simple, pragmatic, therapeutic strategy evaluating pure icosapent ethyl (IPE) at initially higher doses intended to reduce the hospitalization rate and complications in patients with a positive diagnosis of COVID-19.	Completed. 2000 participants, 40 years or older; enrolled no more than 7 days from onset of symptoms and without clear indication of hospitalization
NCT04335032 S.L.A. Pharma AG. Phase 3 [41]	EPA-Free Fatty Acid 500 mg gastro-resistant capsules twice daily (2 g daily) for 28 days	A Randomised, Double-blind, Placebo-Controlled Study of Eicosapentaenoic Acid (EPA-FFA) Gastroresistant Capsules to Treat Hospitalised Subjects with Confirmed COVID-19 (SARS-CoV-2). Treatment failure is defined as the additional or alternative treatment required, or intubation and invasive ventilation, or transfer to the intensive care unit, or death. Reduction of CRP, IL-6, pro-inflammatory cytokines, and chemokines, as well as increased IFN-γ, will be determined.	Recruiting. Estimated enrollment: 284 participants, 18 years and older.
NCT04357990 National Hospital of Iceland (Landspítali). Not applicable Phase [42]	Viruxal Oral and Nasal Spray (Class I CE marked medical device, by Kerecis hf, containing Omega-3 Viruxide) for 14 days.	KONS-COVID-19: Viruxal Oral and Nasal Spray, for Treating the Symptoms of COVID-19. Patients will have their symptoms recorded until no further symptoms are reported, up to a maximum of 28 days follow-up.	Recruiting. Estimated enrollment: 128 participants with mild to moderate symptoms of COVID-19
IRCT20151226025699N3 National Nutrition and Food Technology Research Institute, Iran [43]	1 capsule of omega-3 daily, produced by Omid persina Damavand company, Iran (1000 mg Omega-3-DHA and EPA for each capsule, containing of EPAs+DHAs), for 14 days	The effect of omega-3 supplementation on inflammatory and biochemical markers in critically ill patients with COVID-19 a randomized clinical trial. WBC, Neutrophils, Lymphocytes, LDH, CPK, CBC, CRP: 14 days after the intervention will be determined.	Completed. 50 critically ill COVID-19 patients, from 35 to 85 years old.
NCT04836052 Hamad Medical Corporation (HMC). Phase 3 [44]	Omega-3-oil 2 g PO/NGT/ OGT twice daily for 28 days	Omega-3 Oil Use in COVID-19 Patients in Qatar: a Randomized Controlled Trial. Patients admitted to ICU in HMC on any kind of oxygen support will get omega-3-oil 2 g PO/NGT/ OGT twice daily for 28 days or till ICU discharge or till death	Recruiting. Estimated Enrollment: 372 participants, 18 years and older

CBC: complete blood count; CPK: creatinine phosphokinase; CRP: C-reactive protein; DHA: docosahexaenoic acid; EPA-FFA: eicosapentaenoic acid free-fatty acid; EPA: eicosapentaenoic acid; i.v.: intravenous; ICU: intensive care unit; IFN-γ: interferon-γ; IL-6: interleukin-6; IPE: icosapent ethyl; LDH: lactate dehydrogenase; PO/NGT/OGT: PO = *per os*/by mouth/NGT = nasogastric tube/OGT = orogastric tube; WBC: white blood cell.
